# *EGFR* exon 20 insertion variants A763_Y764insFQEA and D770delinsGY confer favorable sensitivity to currently approved EGFR-specific tyrosine kinase inhibitors

**DOI:** 10.3389/fphar.2022.984503

**Published:** 2022-11-08

**Authors:** Guangjian Yang, Yaning Yang, Jiaqi Hu, Haiyan Xu, Shuyang Zhang, Yan Wang

**Affiliations:** ^1^ Department of Respiratory Medicine, Shandong Cancer Hospital and Institute, Shandong First Medical University and Shandong Academy of Medical Sciences, Jinan, Shandong, China; ^2^ Department of Medical Oncology, National Cancer Center/National Clinical Research Center for Cancer/Cancer Hospital, Chinese Academy of Medical Sciences and Peking Union Medical College, Beijing, China; ^3^ Drug Discovery Business Unit, PharmaBlock Sciences (Nanjing), Inc, Nanjing, Jiangsu, China; ^4^ Department of Comprehensive Oncology, National Cancer Center/National Clinical Research Center for Cancer/Cancer Hospital, Chinese Academy of Medical Sciences and Peking Union Medical College, Beijing, China

**Keywords:** EGFR, exon 20 insertion, A763_Y764insFQEA, D770delinsGY, tyrosine kinase inhibitor, D770_N771insSVD, V769_D770insASV

## Abstract

**Background:** The *EGFR* exon 20 insertions (ex20ins) D770_N771insSVD and V769_D770insASV are most frequent in non-small-cell lung cancer (NSCLC) and are associated with intrinsic resistance to currently approved EGFR tyrosine kinase inhibitors (TKIs). A763_Y764insFQEA and D770delinsGY, respectively, account for 3%–8% and 2.0%–4.8% of *EGFR* ex20ins in NSCLC and are associated with a more favorable response to EGFR-specific TKIs as per case reports. The aim of this study was to elucidate the molecular structures of these mutants and their binding affinities to diverse EGFR TKIs and compare the clinical outcomes in NSCLC patients harboring these mutations.

**Methods:** A real-world cohort study was conducted to evaluate and compare the clinical outcomes of EGFR TKIs among NSCLC patients with different *EGFR* ex20ins mutants in response to EGFR TKIs. The structures of A763_Y764insFQEA and D770delinsGY were also analyzed and drug binding simulations were performed.

**Results:** With a median follow-up of 24.0 months, the first-line objective response rate (ORR), disease control rate (DCR), and median progression-free survival (PFS) were, respectively, 0 (0/16), 50.0% (8/16), and 2.07 months (95%CI, 0–6.25) in patients harboring D770_N771insSVD and V769_D770insASV variants and 33.3% (4/12), 83.3% (10/12), and 9.97 months (95%CI, 4.75–15.19) in patients with A763_Y764insFQEA and D770delinsGY variants. There was a significant difference between the PFS of these two subgroups (median, 9.97 vs.2.07 months, HR = 0.33, 95%CI, 0.13–0.85, *p* = 0.02). Similarly, the PFS was significantly longer after second-line treatment with EGFR TKIs in patients harboring A763_Y764insFQEA and D770delinsGY compared to those with other insertions (median, 6.77 vs.2.23 months, HR = 0.14, *p* < 0.001). Computational simulations indicated that A763_Y764insFQEA and D770delinsGY mutants were structurally similar to wild-type EGFR. In contrast, the C-helix and phosphate-binding loop of D770_N771insSVD and V769_D770insASV had shifted into the drug-binding pocket, resulting in significant steric hindrance and a lack of affinity for the currently approved EGFR inhibitors.

**Conclusion:** NSCLC patients harboring A763_Y764insFQEA and D770delinsGY insertions of EGFR are responsive to the currently approved EGFR TKIs as opposed to patients with the D770_N771insSVD and V769_D770insASV variants. Therefore, A763_Y764insFQEA and D770delinsGY should be classified as active mutations among heterogeneous *EGFR* ex20ins subtypes and the carriers can be treated with the suitable EGFR TKIs.

## Introduction

Exon 19 deletions and the L858R substitution in exon 21 are the most frequent epidermal growth factor receptor (*EGFR*)-activating mutations in non-small-cell lung cancer (NSCLC) and are associated with the sensitivity to EGFR tyrosine kinase inhibitors (TKIs), including the first-generation (1st-gen) gefitinib and erlotinib, second-generation (2nd-gen) afatinib and dacomitinib, and third-generation (3rd-gen) osimertinib (Mok et al., 2009; Soria et al., 2018). In addition, other less common *EGFR* mutations (e.g., G719X, S768I, and L861Q) correlate variably with the sensitivity to EGFR TKIs and with a favorable response to 2nd-gen TKI afatinib (Yang et al., 2015). Generally, TKIs are the preferred treatment for NSCLC patients harboring these *EGFR* mutations.

The prevalence of *EGFR*-activating exon 20 insertions (ex20ins) is only 4%–10% in NSCLC patients (Yasuda et al., 2012; Arcila et al., 2013; Oxnard et al., 2013). The elucidation of the crystal structure of the insertion mutant D770_N771insNPG provided the first insights into the mechanism of EGFR activation and ex20ins-mediated resistance to EGFR inhibitors (Yasuda et al., 2013). Subsequent structural and *in vitro* kinetic studies confirmed that ex20ins can lock the EGFR kinase in an active state in the absence of ligand binding as opposed to the other *EGFR* mutations (Yasuda et al., 2013; Robichaux et al., 2018). The vast majority of ex20ins are located in the phosphate-binding loop (P-loop) region following the C-helix, which is the determinant of EGFR kinase activity (Eck and Yun, 2010; Vyse and Huang, 2019). In addition, nearly all the ex20ins of EGFR exhibit significant steric hindrance and a lack of affinity for the currently approved 1st-to-3rd-gen EGFR-specific TKIs, which can be attributed to the prominent shift of the C-helix and P-loop into the drug-binding pocket of EGFR (Wu et al., 2008; Yasuda et al., 2012; Yasuda et al., 2013; Robichaux et al., 2018; Yang et al., 2020).

Among these heterogeneous ex20ins variants, A763_Y764insFQEA has an insertion within the middle of the C-helix and shows high affinity to diverse EGFR TKIs reported according to both *in vitro* and clinical studies (Arcila et al., 2013; Voon et al., 2013; Yasuda et al., 2013; Hasegawa et al., 2019; Yang et al., 2020; Yang G et al., 2021). NSCLC cells expressing the D770_N771insSVD and V769_D770insASV mutants are resistant to both 1st-gen (IC_50_, 3479.3-5179.7 nM) and 2nd-gen (IC_50_, 54.1-85.9 nM) EGFR TKIs as opposed to those expressing the A763_Y764insFQEA variant (IC_50_, 21.7-33.3 nM to 1st-gen EGFR TKIs; 0.002–0.013 nM to 2nd-gen EGFR TKIs) or the wild-type EGFR (IC_50_, 1127-1333.1 nM to 1st-gen EGFR TKIs; 7.3-39 nM to 2nd-gen EGFR TKIs) (Lee et al., 2019). In addition, another unique insertion D770delinsGY also sensitizes NSCLC cell lines and patients to 2nd- or 3rd-gen EGFR TKIs (Jänne et al., 2011; Kosaka et al., 2017; Yang et al., 2020; Yang J et al., 2021). Codon Asp770 (D770) is located in the pivot point of the EGFR C-helix, and the replacement of D770 with the inserted glycine potentially restores the wild-type C-helix conformational changes and the affinity for TKIs (Kosaka et al., 2017). Given the low frequency of A763_Y764insFQEA (3%–8%) (Riess et al., 2018; Yang et al., 2020; Yang G et al., 2021; Geng et al., 2022) and D770delinsGY (2.0%–4.8%) (Yang et al., 2020; Yang J et al., 2021) variants, their response to diverse EGFR TKIs in the real world NSCLC cohorts is ambiguous. In addition, their structures and binding affinity to EGFR TKIs have also not been ascertained. This highlights the need for further studies to understand the underlying mechanisms underlying the favorable response of NSCLC patients harboring these two specific insertion variants of *EGFR* to EGFR TKIs.

Therefore, we conducted a real-world study to investigate the targeted outcomes of diverse EGFR TKIs in NSCLC patients with the A763_Y764insFQEA and D770delinsGY variants, and compared them with the therapeutic response in patients harboring other *EGFR* ex20ins subtypes. Furthermore, we also constructed the three-dimensional (3D) structures of A763_Y764insFQEA and D770delinsGY to simulate and predict their binding affinity to EGFR TKIs.

## Patients and methods

### Patients

This study retrospectively collected medical data of metastatic NSCLC patients with *EGFR* ex20ins at the National Cancer Center/Cancer Hospital, Chinese Academy of Medical Sciences between 10 May 2016, and 10 September 2021. The inclusion criteria were as follows: 1) stage IV disease at initial diagnosis; 2) ≥18 years of age; 3) histologically or cytologically confirmed NSCLC; 4) *EGFR* ex20ins mutations confirmed at initial diagnosis by next-generation sequencing (NGS) testing with formalin-fixed, paraffin-embedded tissue samples or liquid biopsy samples; and 5) documented treatment with EGFR TKIs in the first- or second-line setting. The NGS testing in this study was performed in institutional laboratories or qualified third-party genetic testing companies as documented in medical records, which had acquired the national quality system certification in China, and all of the NGS tests were carried out based on the Illumina sequencing system. All clinical data were extracted from electronic records. As a retrospective observational study, informed consent was exempted. The study was approved by the Ethics Committee of the National Cancer Center and conducted in accordance with the Declaration of Helsinki.

### Treatment and assessment

Eligible patients were included in molecular and efficacy analysis. Patients treated with EGFR TKIs were at a standard dose according to clinical guidelines in practice. Baseline images of measurable target lesions were obtained with computed tomography of the chest and abdomen, and magnetic resonance imaging of the brain. Guideline from the Response Evaluation Criteria in Solid Tumors version 1.1 (RECIST 1.1) was performed to identify a response of complete response (CR), partial response (PR), stable disease (SD), or progressive disease (PD). The targeted response was evaluated by investigators involved in this study and had been double-checked according to the medical images provided by patients. Progression-free survival (PFS) was defined as the time from initiation of EGFR TKIs to the date of documented disease progression or death from any cause. The objective response rate (ORR) was calculated as the percentage of confirmed CR and PR. Disease control rate (DCR) was defined as the percentage of CR, PR, and SD.

### Molecular dynamics simulation

The 3D-modeling of A763_Y764insFQEA and D770delinsGY mutants were computationally constructed based on the crystal structure of the wild-type (WT) EGFR kinase domain using the Schrödinger software (2020-1 Release) (PDB: 4I23). The protein was prepared using Maestro software (Schrödinger 2021-1 Release) in the Schrödinger modeling package. Compounds of afatinib, dacomitinib, osimertinib, poziotinib, mobocertinib (TAK-788), and CLN-081 (TAS6417) were constructed using the 3D-sketcher module in Maestro. For the prediction of bioactive conformation and binding modes with the abovementioned compounds, we conducted docking simulations using the GLIDE (Schrödinger 2020-1 Release) program from Schrödinger Inc. (Portland, Oregon). The computer-based binding free energy (ΔG_bind_) was calculated with the GlideScore method and the Molecular Mechanics/Generalized Born Surface Area (MM/GBSA) method. Figures were produced by Molecular Operating Environment software (MOE 2021.01 Chemical Computing Group, Montreal, Canada). The detailed content regarding ΔG_bind_ of a protein–ligand complex is listed in the Supplementary Appendix.

### Statistical analysis

Statistical analyses were conducted using SPSS version 23.0 (SPSS Inc., Chicago, IL, United States). Continuous variables were summarized using medians and ranges, and categorical variables were described using frequency and percentage. Comparison among the subgroups was performed using analysis of variance or Chi-Square test accordingly. PFS and OS were analyzed using the Kaplan–Meier method. PFS between different subgroups were compared using a log-rank test (two-sided), and the corresponding hazard ratio (HR) and 95% confidence interval (CI) were estimated using the Cox proportional regression model. *p* < 0.05 was considered statistically significant. *p* values for these analyses are nominal, and all are two-sided. Survival curves were plotted using the R software (version 3.6.3).

## Results

### Patient characteristics

As the flow chart showed (Supplementary Figure), 35 and 24 eligible patients who had received diverse EGFR TKIs as first- or second-line therapy were respectively included. In terms of first-line targeted therapy, 48.6% (*n* = 17) were females, 57.1% (*n* = 20) were never-smokers, 94.3% (*n* = 33) were lung adenocarcinomas, and 22.9% (*n* = 8) presented with baseline central nervous system (CNS) metastases. Nearly all of the patients (*n* = 34, 97.1%) received NGS testing *via* tumor tissues. The molecular findings revealed that 22.9% (*n* = 8) harbored ex20ins of A763_Y764insFQEA, 11.4% (*n* = 4) with D770delinsGY, 25.7% (*n* = 9) with D770_N771insSVD, 20.0% (*n* = 7) with V769_D770insASV, and the others (20.0%, *n* = 7) carried less common ex20ins variants including P772_H773insGHP, D770_N771insGD, N771_P772insH, P772_H773insH, H773_V774insAH, and H773delinsRY. Baseline clinicopathological characteristics of *EGFR* ex20ins patients with the first-line therapy are demonstrated in Table 1. Similarly, second-line targeted therapy data are listed in the Supplementary Table.

**TABLE 1:** Clinicopathological characteristics of NSCLC patients with EGFR exon 20 insertions treated with first-line targeted therapy.

Characteristics	FQEA/GY (*n* = 13)	Others (*n* = 11)	Overall (*n* = 24)
Age (years)	53.1 ± 9.4	57.4 ± 6.3	55.0 ± 8.2
Gender			
Male	4 (30.8%)	3 (27.3%)	7 (29.2%)
Female	9 (69.2%)	8 (72.7%)	17 (70.8%)
Pathology			
Adenocarcinoma	11 (84.6%)	11 (100.0%)	22 (91.6%)
Adenosquamous carcinoma	1 (7.7%)	0 (0)	1 (4.2%)
Squamous carcinoma	1 (7.7%)	0 (0)	1 (4.2%)
Smoking history			
Never	11 (84.6%)	8 (72.7%)	19 (79.2%)
Current/former	2 (15.4%)	3 (27.3%)	5 (20.8%)
CNS metastases			
Absence	8 (61.5%)	9 (81.8%)	17 (70.8%)
Presence	5 (38.5%)	2 (18.2%)	7 (29.2%)
Liver metastases			
Absence	12 (92.3%)	11 (100.0%)	23 (95.8%)
Presence	1 (7.7%)	0 (0)	1 (4.2%)
NGS specimen			
Tumor tissue	12 (92.3%)	10 (90.9%)	22 (91.7%)
Plasma	1 (7.7%)	1 (9.1%)	2 (8.3%)
TP53 mutation			
None	3 (23.1%)	1 (9.1%)	4 (16.7%)
Yes	2 (15.4%)	6 (54.5%)	8 (33.3%)
NA	8 (61.5%)	4 (36.4%)	12 (50.0%)

FQEA, A763_Y764insFQEA; GY, D770delinsGY; NA, not available.

^†^There were no differences among the subgroups.

### Efficacy

The cutoff time of this study was 15 October 2021, with a median follow-up of 24.0 months (95%CI, 19.49–28.51 months). All 35 patients had PFS events in the first-line setting, and 22 patients (91.7%) had PFS events in the second-line setting.

In the first-line setting, among seven patients harboring V769_D770insASV, 4 showed *de novo* resistance to 1st- or 2nd-gen EGFR TKIs. Another two patients showed SD, with PFS of 5.6 and 7.7 months, and one case revealed SD but with a much longer PFS (35.9 months) to afatinib. Nine patients carried D770_N771insSVD and 6 of them showed primary drug resistance to 1st- or 3rd-gen EGFR TKIs. Another three cases all revealed SD, with PFS ranging between 3.8 and 5.8 months. The overall ORR, DCR, and median PFS (mPFS) was 0 (0/16), 50.0% (8/16), and 2.07 months (95%CI, 0–6.25) for patients with V769_D770insASV and D770_N771insSVD (ASV/SVD) mutants.

A total of 12 patients were identified to be A763_Y764insFQEA and D770delinsGY mutants (FQEA/GY). Among 8 patients with A763_Y764insFQEA, only one showed *de novo* resistance to 1st-gen EGFR TKI (icotinib). Two patients showed PR, including one with afatinib (PFS, 24 months) and one with osimertinib (PFS, 3.1 months). The other 5 patients all kept SD from 1st- or 3rd-gen EGFR TKIs, with PFS ranging between 3.4 and 15.3 months. Among four patients with D770delinsGY, two showed PR and one showed SD to osimertinib, with PFS of 5.1 and 18.2 months. Only one case revealed *de novo* resistance to 3rd-gen EGFR TKI (almonertinib). The overall ORR, DCR, and mPFS for patients carrying FQEA/GY mutants were 33.3% (4/12), 83.3% (10/12), and 9.97 months (95%CI, 4.75–15.19).

**FIGURE 1:**
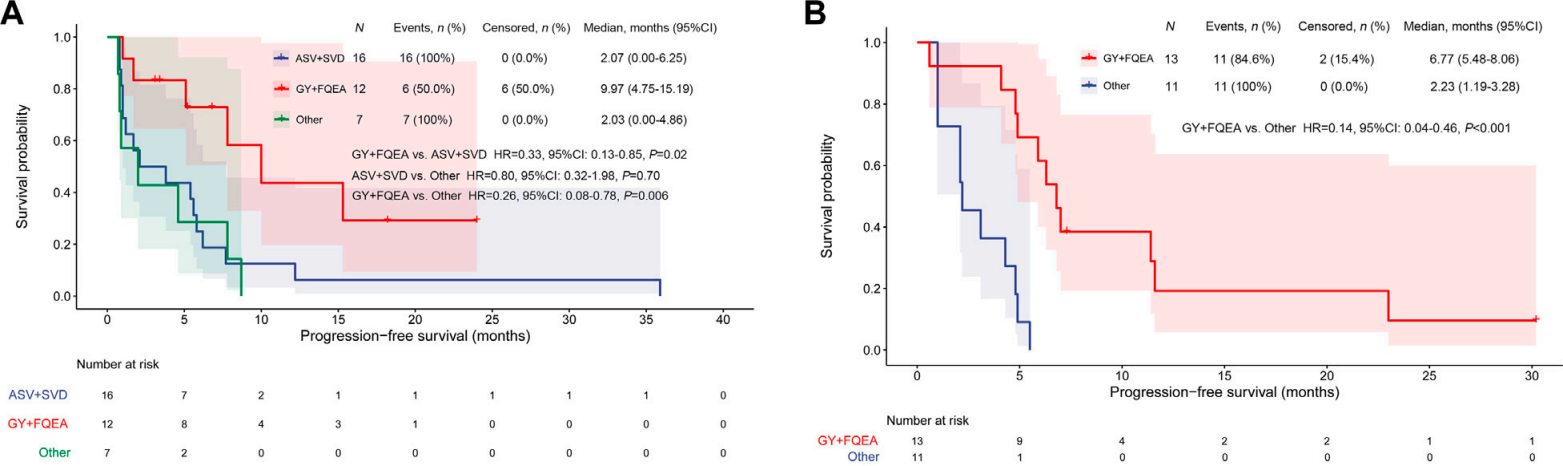
Kaplan–Meier curves for PFS by EGFR TKI targeted therapy among different ex20ins patients in the first-line **(A)** and second-line setting **(B)**.

In addition, 7 patients harbored other less common ex20ins subtypes, including P772_H773insGHP (*n* = 1), D770_N771insGD (*n* = 1), N771_P772insH (*n* = 1), P772_H773insH (*n* = 2), H773_V774insAH (*n* = 1), and H773delinsRY (*n* = 1), and the respective ORR, DCR, and mPFS were 0 (0/7), 42.9% (3/7), and 2.03 months (95%CI, 0–4.86) for them receiving 1st-to-3rd-gen EGFR TKIs. It was observed significant mPFS difference between subgroup FQEA/GY vs. ASV/SVD mutants (9.97 vs.2.07 months, HR = 0.33, 95%CI, 0.13–0.85, *p* = 0.02), and FQEA/GY vs. other insertion mutants (9.97 vs.2.03 months, HR = 0.26, 95%CI, 0.08–0.78, *p* = 0.006). No difference on mPFS was observed between subgroup ASV/SVD and other insertion mutants (2.07 vs.2.03 months, HR = 0.80, 95%CI, 0.32–1.98, *p* = 0.70). The PFS on the abovementioned three insertion subgroups was listed in Figure 1A. The targeted activity of EGFR TKIs for NSCLC patients with diverse ex20ins variants in the first-line setting was presented in Table 2.

**TABLE 2:** First-line EGFR TKIs for NSCLC patients with diverse *EGFR* exon 20 insertion subtypes (*n* = 35).

Exon 20 insertion variant	TKI	Best response	PFS (months)	Ongoing treatment
FQEA/GY (*n* = 12)				
A763_Y764insFQEA	Icotinib	PD	1.0	No
A763_Y764insFQEA	Osimertinib	PR	3.1	Yes
A763_Y764insFQEA	Osimertinib	SD	3.4	Yes
A763_Y764insFQEA	Osimertinib	SD	5.2	Yes
A763_Y764insFQEA	Dacomitinib	SD	6.8	Yes
A763_Y764insFQEA	Osimertinib	SD	7.8	No
A763_Y764insFQEA	Gefitinib	SD	15.3	No
A763_Y764insFQEA	Afatinib	PR	24.0	Yes
D770delinsGY	Almonertinib	PD	1.7	No
D770delinsGY	Osimertinib	PR	5.1	No
D770delinsGY	Osimertinib	SD	10.0	No
D770delinsGY	Osimertinib	PR	18.2	Yes
ASV/SVD (*n* = 16)				
V769_D770insASV	Erlotinib	PD	0.9	No
V769_D770insASV	Gefitinib	PD	1.0	No
V769_D770insASV	Afatinib	PD	1.2	No
V769_D770insASV	Gefitinib	PD	1.7	No
V769_D770insASV	Gefitinib	SD	5.6	No
V769_D770insASV	Osimertinib	SD	7.7	No
V769_D770insASV	Afatinib	SD	35.9	No
D770_N771insSVD	Gefitinib	PD	0.8	No
D770_N771insSVD	Osimertinib	PD	0.8	No
D770_N771insSVD	Gefitinib	PD	1.0	No
D770_N771insSVD	Gefitinib	PD	1.3	No
D770_N771insSVD	Osimertinib	SD	3.8	No
D770_N771insSVD	Afatinib	SD	5.4	No
D770_N771insSVD	Osimertinib	SD	5.8	No
D770_N771insSVD	Osimertinib	SD	6.2	No
D770_N771insSVD	Osimertinib	SD	12.2	No
Other insertions (*n* = 7)				
P772_H773insH	Osimertinib	PD	0.7	No
P772_H773insGHP	Gefitinib	PD	0.8	No
H773delinsRY	Osimertinib	PD	0.9	No
D770_N771insGD	Afatinib	PD	2.0	No
H773_V774insAH	Osimertinib	SD	4.6	No
N771_P772insH	Afatinib	SD	7.8	No
P772_H773insH	Afatinib	SD	8.7	No

PD, progressive disease; PFS, progression-free survival; PR, partial response; SD, stable disease; TKI, tyrosine kinase inhibitor.

In the second-line setting, seven patients were detected to be A763_Y764insFQEA. Among them, 3 showed PR and 4 revealed SD to 2nd- or 3rd-gen EGFR TKIs, with PFS outcomes between 4.8 and 30.2 months. Among six patients harboring D770delinsGY, one showed *de novo* resistance to afatinib (PFS, 0.6 months). Two patients showed PR, and 3 showed SD to 2nd- or 3rd-gen EGFR TKIs (afatinib, dacomitinib, osimertinib), with PFS between 4.1 and 7.3 months. The ORR, DCR, and mPFS were 38.5% (5/13), 92.3% (12/13), and 6.77 months (95%CI, 5.48–8.06) for patients with FQEA/GY mutants. In addition, only a total of 6 patients harbored ASV/SVD mutants and showed short PFS (1.0–5.5 months) and no response treated with 2nd- or 3rd-gen EGFR TKIs. In addition, 5 patients harbored other ex20ins subtypes (3 of H773_V774insNPH, one of H773_V774insPH, and one of N771delinsNPHVC). Among these non-FQEA/GY patients, the ORR, DCR, and mPFS were 9.1% (1/11), 63.6% (7/11), and 2.23 months (95%CI, 1.19–3.28), respectively. The mPFS of patients harboring FQEA/GY mutants was significantly longer compared with those with other insertions (6.77 vs.2.23 months, HR = 0.14, 95%CI, 0.04–0.46, *p* < 0.001) (Figure 1B).

### Molecular structures and binding activity to EGFR TKIs

**FIGURE 2:**
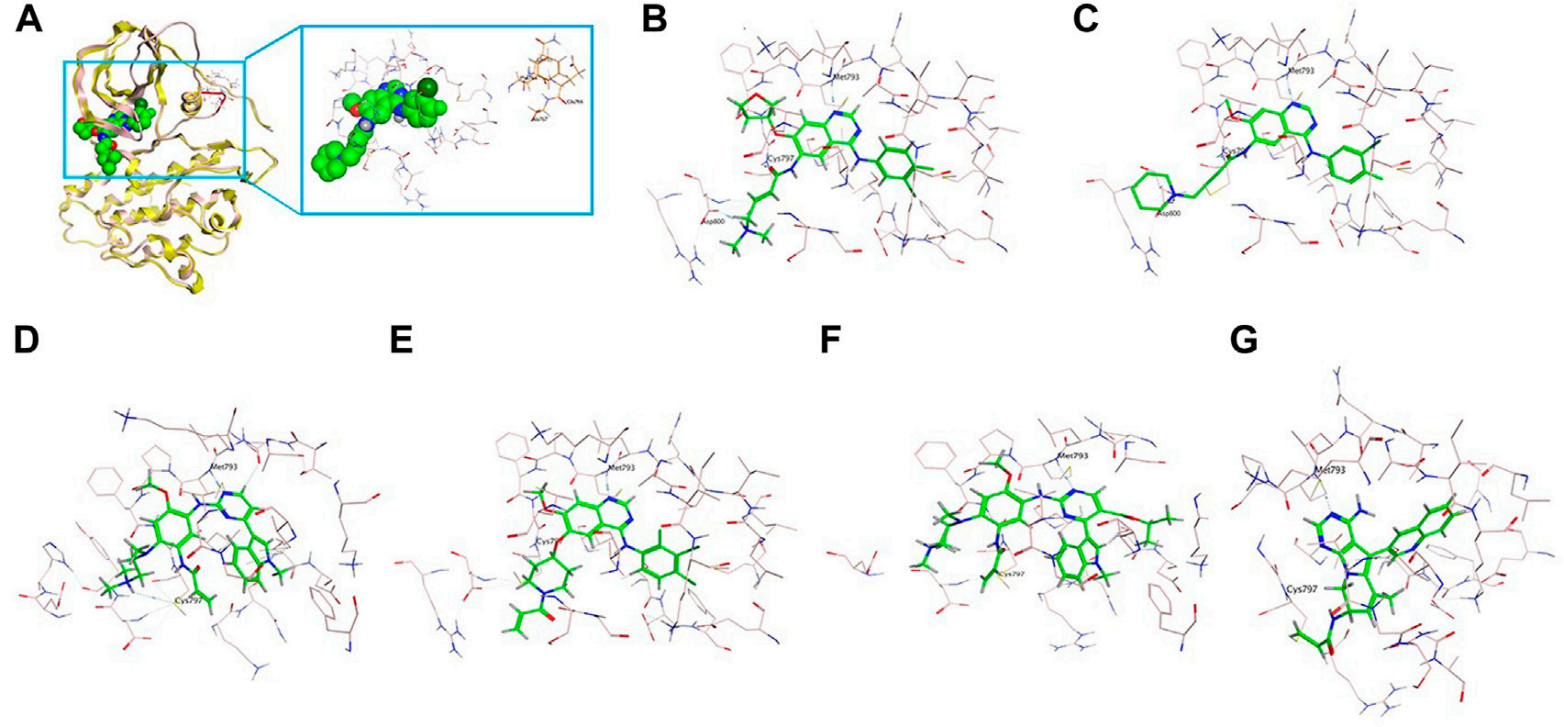
3D-based structure of A763_Y764insFQEA variant **(A)** with the binding pocket (marked in pink) of a certain inhibitor (marked in green) and the inserted amino acids FQEA (marked in brown). Binding modes of diverse EGFR TKIs including afatinib **(B)**, dacomitinib **(C)**, osimertinib **(D)**, poziotinib **(E)**, mobocertinib **(F)**, and CLN-081 **(G)** for the A763_Y764insFQEA variant.

On the 3D-based structure of the A763_Y764insFQEA variant, although the conformation was slightly altered by the insertion of four amino acids Phe-Gln-Glu-Ala within the C-helix (marked in red in Figure 2A), the overall protein structure of EGFR keeps stable and the binding affinity of small molecules is not affected. It is observed that there is a certain distance around the binding pocket (marked in pink) of the small molecule (marked in green) and the inserted FQEA sequence (marked in brown), indicating no direct effect on the binding of EGFR inhibitors (Figure 2A). The 2nd-gen TKI afatinib (Figure 2B) and dacomitinib (Figure 2C) both reveal the most favorable binding affinity to A763_Y764insFQEA mutant by the interaction of H-bonds between N of quinazoline-Met793 and Carbonyl-Cys797, along with salt-bridge between Amine-Asp800, the covalent bond between Michael acceptor-Cys797, and hydrophobic interaction with 3-chloro-4-fluorophenyl. By comparison, 3rd-gen TKI osimertinib (Figure 2D), and novel anti-ex20ins inhibitors mobocertinib (Figure 2F) and CLN-081 (Figure 2G) bind to A763_Y764insFQEA mutant with less potency, interacting with H-bond between N of pyrimidine-Met793 and covalent bond between Michael acceptor-Cys797. In addition, poziotinib also interacts better with A763_Y764insFQEA confirmation by H-bond between N of pyrimidine-Met793, the covalent bond between Michael acceptor-Cys797 and hydrophobic interaction with 2-fluoro-3-chloro-4- fluorophenyl (Figure 2E).

**FIGURE 3:**
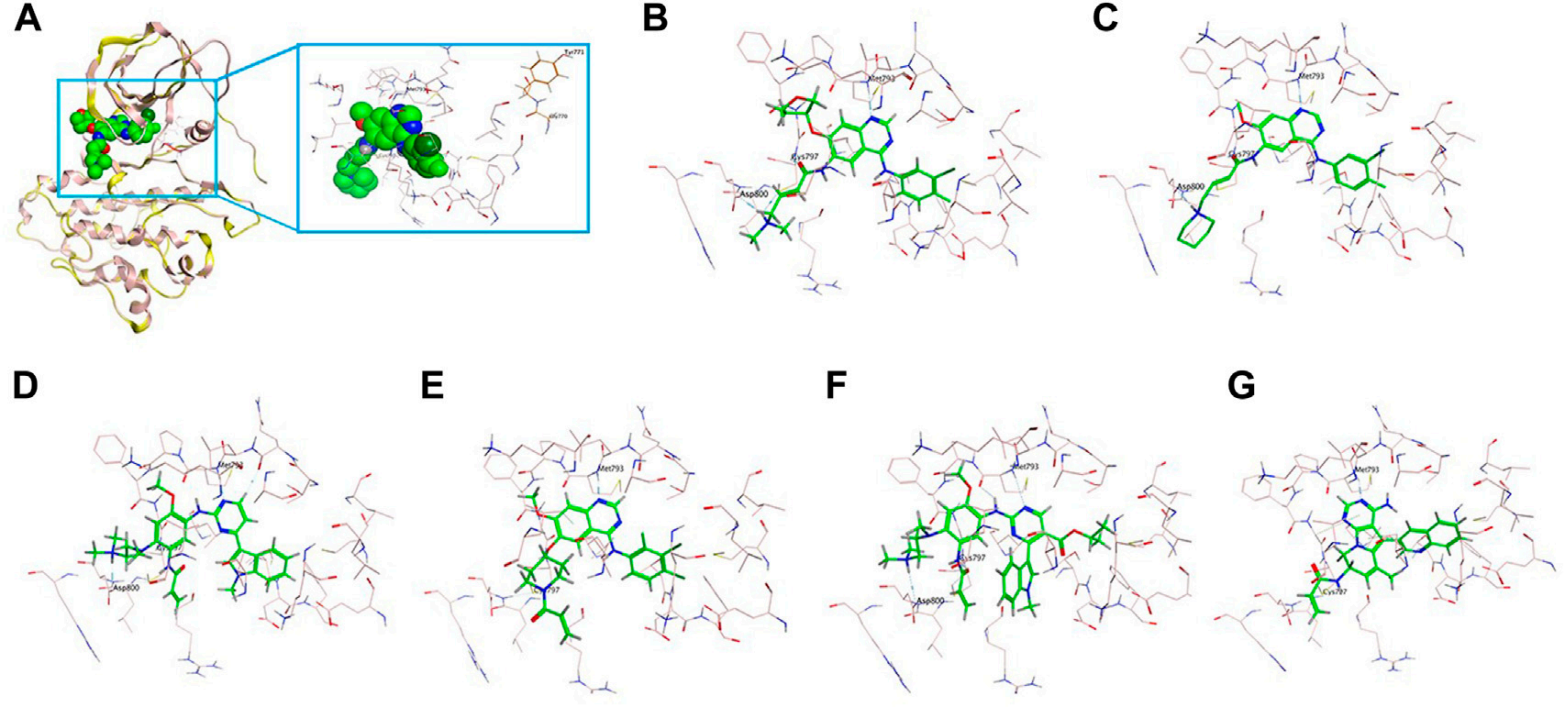
3D-based structure of G770delinsGY variant **(A)** with the binding pocket (marked in pink) of a certain inhibitor (marked in green) and the inserted amino acids GY (marked in brown). Binding modes of diverse EGFR TKIs including afatinib **(B)**, dacomitinib **(C)**, osimertinib **(D)**, poziotinib **(E)**, mobocertinib **(F)** and CLN-081 **(G)** for the D770delinsGY variant.

Similarly, the insertion of two amino acids Gly-Tyr (GY) at codon D770 after the C-helix (marked in red in Figure 3A) as well mildly altered the conformation, but the overall protein structure of EGFR has not been affected, with the binding affinity of small molecules not affected. It is also observed that the binding pocket (marked in pink) of the small molecule (marked in green) is near the inserted GY sequence (marked in brown), but still with a certain distance on the TKI binding (Figure 3A). Afatinib (Figure 3B) and dacomitinib (Figure 3C) reveal the most potent binding activity to the D770delinsGY variant by the interaction of H-bonds between N of quinazoline-Met793 and Carbonyl-Cys797, along with salt-bridge between Amine-Asp800, the covalent bond between Michael acceptor-Cys797, and hydrophobic interaction with 3-chloro-4-fluorophenyl. Osimertinib (Figure 3D) and mobocertinib (Figure 3F) interact with the D770delinsGY variant less potently by H-bond between N of pyrimidine-Met793, the covalent bond between Michael acceptor-Cys797, and salt-bridge between Amine-Asp800. Poziotinib interacts with the D770delinsGY variant by N of quinazoline-Met793, the covalent bond between Michael acceptor-Cys797, and hydrophobic interaction with 2-fluoro-3-chloro-4- fluorophenyl (Figure 3E). CLN-081 shows less interaction with the H-bond between N of quinazoline-Met793 and the covalent bond between Michael acceptor-Cys797 (Figure 3G). ΔG_bind_ of EGFR TKIs discussed above for the two specific ex20ins variants were calculated by dynamics simulations and listed in Table 3.

**TABLE 3:** Binding free energy (kcal/mol) of different EGFR inhibitors for A763_Y764insFQEA and D770delinsGY insertions.

EGFR inhibitor	A763_Y764insFQEA		D770delinsGY	
	GlideScore	MM/GBSA	GlideScore	MM/GBSA
Afatinib	−9.177	−88.75	−9.524	−97.02
Dacomitinib	−9.088	−86.21	−9.121	−89.67
Osimertinib	−8.082	−83.28	−8.786	−89.69
Poziotinib	−8.418	−96.37	−8.896	−96.38
Mobocertinib	−8.280	−81.84	−7.188	−85.27
CLN-081	−7.916	−72.86	−7.067	−69.06

MM/GBSA, Molecular Mechanics/Generalized Born Surface Area.

## Discussion

In this study, we provided comprehensive evidence for the targeted response of diverse EGFR TKIs among heterogeneous ex20ins EGFR mutants to diverse EGFR TKIs in a real-world NSCLC cohort, along with structural insights into the binding modes and binding activities of the A763_Y764insFQEA and D770delinsGY variants in particular. Our study is currently the largest in terms of the sample size that analyzes the response of ex20ins subtypes A763_Y764insFQEA and D770delinsGY to EGFR TKIs.

D770_N771insSVD and V769_D770insASV are the most common ex20ins mutants that are intrinsically resistant to 1st-to-3rd-gen EGFR TKIs (Naidoo et al., 2015; Riess et al., 2018; Yang et al., 2020; Yang G et al., 2021). Consistent with this, we did not observe any difference in the PFS of patients harboring ASV/SVD insertions and those with other variants (median, 2.07 vs.2.03 months, HR = 0.80, *p* = 0.70) following EGFR-TKIs as the first-line treatment. The currently approved 1st-to-3rd-gen EGFR TKIs have overall limited activity against ex20ins variants of EGFR, with general ORRs of 8.7%–11%, and PFS ranging between 2.4 and 2.7 months (Yasuda, et al., 2012; Yasuda et al., 2013; Beau-Faller et al., 2014; Naidoo et al., 2015; Chen et al., 2016; Kuiper et al., 2016; Kosaka et al., 2017; Tu et al., 2017; Byeon et al., 2019; Hasegawa et al., 2019). Our previous study on nationwide data also indicated an ORR of 8.7%, and a shorter mPFS of 2.9 months for patients with *EGFR* ex20ins (Yang et al., 2020) in response to EGFR TKIs as first-line therapy. The patients who received 1st-gen EGFR TKIs in the first-line setting showed no response, with a mPFS of only 2.0 months (Yang et al., 2020).

In addition, we observed a more improved ORR (33.3% vs. 0) and significant PFS benefit (median, 9.97 vs. 2.07 months, HR = 0.33, *p* = 0.02) in patients harboring FQEA/GY mutants compared to those with ASV/SVD treated with 1st-to-3rd-gen EGFR TKIs as the first-line therapy. Similarly, an improved ORR (38.5% vs. 9.1%) and significant PFS benefit (median, 6.77 vs. 2.23 months, HR = 0.14, *p* < 0.001) were observed in patients with FQEA/GY mutants compared with those harboring other insertions in a second-line setting. The A763_Y764insFQEA variant is generally considered to be a specific ex20ins subtype to be sensitive to EGFR TKIs (Arcila et al., 2013; Voon et al., 2013; Yasuda et al., 2013; Hasegawa et al., 2019). In our previous study, two patients carrying A763_Y764insFQEA obtained reasonable PFS benefits when treated with afatinib (PFS of 8.2 months) and erlotinib (PFS of 14.3 months) (Yang et al., 2020). In another study by our group, the mPFS of NSCLC patients with *EGFR* ex20ins treated with osimertinib was 2.3 months. The mPFS of patients with FQEA/GY insertion was longer than those with other variants (4.2 vs. 2.2 months, *p* = 0.164), and half of the FQEA/GY patients showed PR to osimertinib (Yang G et al., 2021). Taken together, the A763_Y764insFQEA and D770delinsGY variants might be more responsive to the currently approved EGFR TKIs compared to the other ex20ins subtypes, and they should be classified as such in the clinical setting.

Yasuda et al. (2013) established A763_Y764insFQEA as an EGFR TKI-sensitizing insertion and showed that the insertion of FQEA shifted the C-helix and altered the length of the β3-αC loop leading to an I759A replacement, which resulted in catalytic activation. Kosaka et al. (2017) generated Ba/F3 cells expressing D770delinsGY-mutant EGFR and found that this recombinant cell line was significantly more sensitive to dacomitinib compared to the other ex20ins Ba/F3 cell lines (*p* = 0.013). In addition, the D770delinsGY-expressing cells were slightly more sensitive to afatinib while other ex20ins conferred resistance to neratinib. Jänne et al. (2011) reported the case of one NSCLC patient with D770delinsGY who achieved a dramatic and sustained response to dacomitinib. Subsequently, Kosaka et al. (2017) demonstrated that the presence of glycine at codon 770 in D770delinsGY restored the sensitivity to TKIs, either by removing the steric hindrance imposed by D770, or by simply increasing the flexibility of the inserted loop. However, these initial studies did not evaluate the impact of these two specific insertions on the structure of EGFR and their binding affinities to diverse EGFR TKIs. In this study, we first constructed *in silico* structures of A763_Y764insFQEA and D770delinsGY and found that neither insertion alters the overall EGFR protein structure and drug binding pocket. The binding pocket was still located at a certain distance from the inserted FQEA or GY sequence and therefore did not exert any steric effect on the binding to EGFR TKIs. In addition, FQEA/GY mutants were most responsive and showed optimum binding to the 2nd-gen TKIs afatinib and dacomitinib.

**FIGURE 4:**
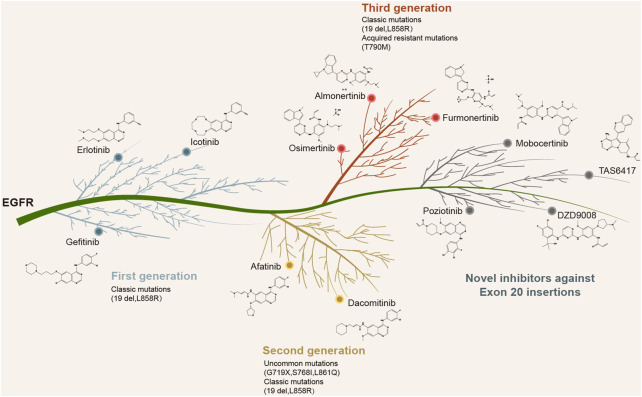
Currently approved or newly designed EGFR inhibitors against *EGFR* classic mutations, uncommon alterations, and exon 20 insertions.

Recently, the EGFR/MET-bispecific monoclonal antibody amivantamab was approved by the Food and Drug Administration (FDA) for NSCLC patients with *EGFR* ex20ins, based on an ORR of 40% and a mPFS of 8.3 months (Park et al., 2021). In addition, other newly designed EGFR kinase inhibitors including CLN-081, mobocertinib, and sunvozertinib (DZD9008) have also demonstrated selectivity against *EGFR* ex20ins (Piotrowska et al., 2021; Prelaj et al., 2021; Riely et al., 2021; Wang et al., 2022). Mobocertinib received FDA approval for NSCLC patients harboring *EGFR* ex20ins who had progressed following platinum-based chemotherapy, based on an ORR of 28% and a mPFS of 7.3 months (Zhou et al., 2021). A recent study on poziotinib revealed a confirmed ORR of 32% and a mPFS of 5.5 months in NSCLC patients with *EGFR* ex20ins (Elamin et al., 2022). Furthermore, the sensitivity to poziotinib was highly dependent on the insertion location. The near-loop insertions (amino acids A767-P772) were associated with a greater sensitivity compared to the far-loop insertions (H773-C775), with respective ORRs of 46% and 0% observed in near vs. far-loop, respectively (Elamin et al., 2022). The blueprint of precision-targeted therapy for NSCLC patients harboring the classic mutations, uncommon alterations, and ex20ins in EGFR is shown in Figure 4 **.** Several targeted inhibitors of *EGFR* ex20ins variants are currently in development and are expected to improve the therapeutic outcomes in NSCLC patients.

Although we have comprehensively analyzed the clinical activity of the currently approved EGFR TKIs for the specific *EGFR* ex20ins variants A763_Y764insFQEA and D770delinsGY in advanced NSCLC patients and provided evidence from structural and molecular dynamics simulation, several limitations must be noted. First, the retrospective nature of the real-world study likely introduced selection bias. In addition, although patients with FQEA/GY insertions represented the largest group in our cohort, their number was still small, which limits the reliability of the results regarding the clinical efficacy of the EGFR TKIs. Finally, although we explained the potential mechanism with respect to the binding affinity of the ex20ins variants, the structural modeling, and computational simulation will have to be validated by studies on cell lines and patient-derived xenograft models.

In conclusion, NSCLC patients harboring the A763_Y764insFQEA and D770delinsGY ex20ins variants of EGFR display a favorable response to currently approved EGFR TKIs, which should be classified according to the ex20ins variants for more effective therapeutic responses.

## Data Availability

Data supporting the results presented in this study are available from the corresponding author upon reasonable request.

## Abbreviations

CI, confidence interval; CNS, central nervous system; CR, complete response; DCR, disease control rate; EGFR, epidermal growth factor receptor; ex20ins, exon 20 insertions; HR, hazard ratio; MM/GBSA, Molecular Mechanics/Generalized Born Surface Area; NGS, next generation sequencing; NSCLC, non-small-cell lung cancer; ORR, objective response rate; OS, overall survival; RECIST, Response Evaluation Criteria in Solid Tumors; PD, progressive disease; PFS, progression-free survival; P-loop, phosphate-binding loop; PR, partial response; SD, stable disease; TKIs, tyrosine kinase inhibitors; WT, wild type; ΔGbind, binding free energy; 1st-gen, first-generation; 2nd-gen, second-generation; 3D, three-dimensional; 3rd-gen, third-generation.
