# Supplementary material for: EGFR exon 20 insertion variants A763_Y764insFQEA and D770delinsGY confer favorable sensitivity to currently approved EGFR-specific tyrosine kinase inhibitors
Source: Front Pharmacol. 2022 Nov 8;13:984503. doi: 10.3389/fphar.2022.984503 (PMC9679652; doi:10.3389/fphar.2022.984503)
Supplement: Supplementary file 1 [file Table1.docx]

**Clinicopathological characteristics of NSCLC patients with *EGFR* exon 20 insertions treated with second-line targeted therapy.**

| **Characteristics** | **FQEA/GY (n=13)** | **Others (n=11)** | **Overall (n=24)** |
| --- | --- | --- | --- |
| **Age (years)** | 53.1±9.4 | 57.4±6.3 | 55.0±8.2 |
| **Gender** |  |  |  |
| Male | 4 (30.8%) | 3 (27.3%) | 7 (29.2%) |
| Female | 9 (69.2%) | 8 (72.7%) | 17 (70.8%) |
| **Pathology** |  |  |  |
| Adenocarcinoma | 11 (84.6%) | 11 (100.0%) | 22 (91.6%) |
| Adeno-squamous carcinoma | 1 (7.7%) | 0 (0) | 1 (4.2%) |
| Squamous carcinoma | 1 (7.7%) | 0 (0) | 1 (4.2%) |
| **Smoking History** |  |  |  |
| Never | 11 (84.6%) | 8 (72.7%) | 19 (79.2%) |
| Current/former | 2 (15.4%) | 3 (27.3%) | 5 (20.8%) |
| **CNS metastases** |  |  |  |
| Absence | 8 (61.5%) | 9 (81.8%) | 17 (70.8%) |
| Presence | 5 (38.5%) | 2 (18.2%) | 7 (29.2%) |
| **Liver metastases** |  |  |  |
| Absence | 12 (92.3%) | 11 (100.0%) | 23 (95.8%) |
| Presence | 1 (7.7%) | 0 (0) | 1 (4.2%) |
| **NGS specimen** |  |  |  |
| Tumor tissue | 12 (92.3%) | 10 (90.9%) | 22 (91.7%) |
| Plasma | 1 (7.7%) | 1 (9.1%) | 2 (8.3%) |
| **TP53 mutation** |  |  |  |
| None | 3 (23.1%) | 1 (9.1%) | 4 (16.7%) |
| Yes | 2 (15.4%) | 6 (54.5%) | 8 (33.3%) |
| NA | 8 (61.5%) | 4 (36.4%) | 12 (50.0%) |

FQEA, A763_Y764insFQEA. GY, D770delinsGY. NA, not available.

^†^There were no differences among the subgroups.
