# Supplementary figures and images for: EGFR exon 20 insertion variants A763_Y764insFQEA and D770delinsGY confer favorable sensitivity to currently approved EGFR-specific tyrosine kinase inhibitors
Source: Front Pharmacol. 2022 Nov 8;13:984503. doi: 10.3389/fphar.2022.984503 (PMC9679652; doi:10.3389/fphar.2022.984503)

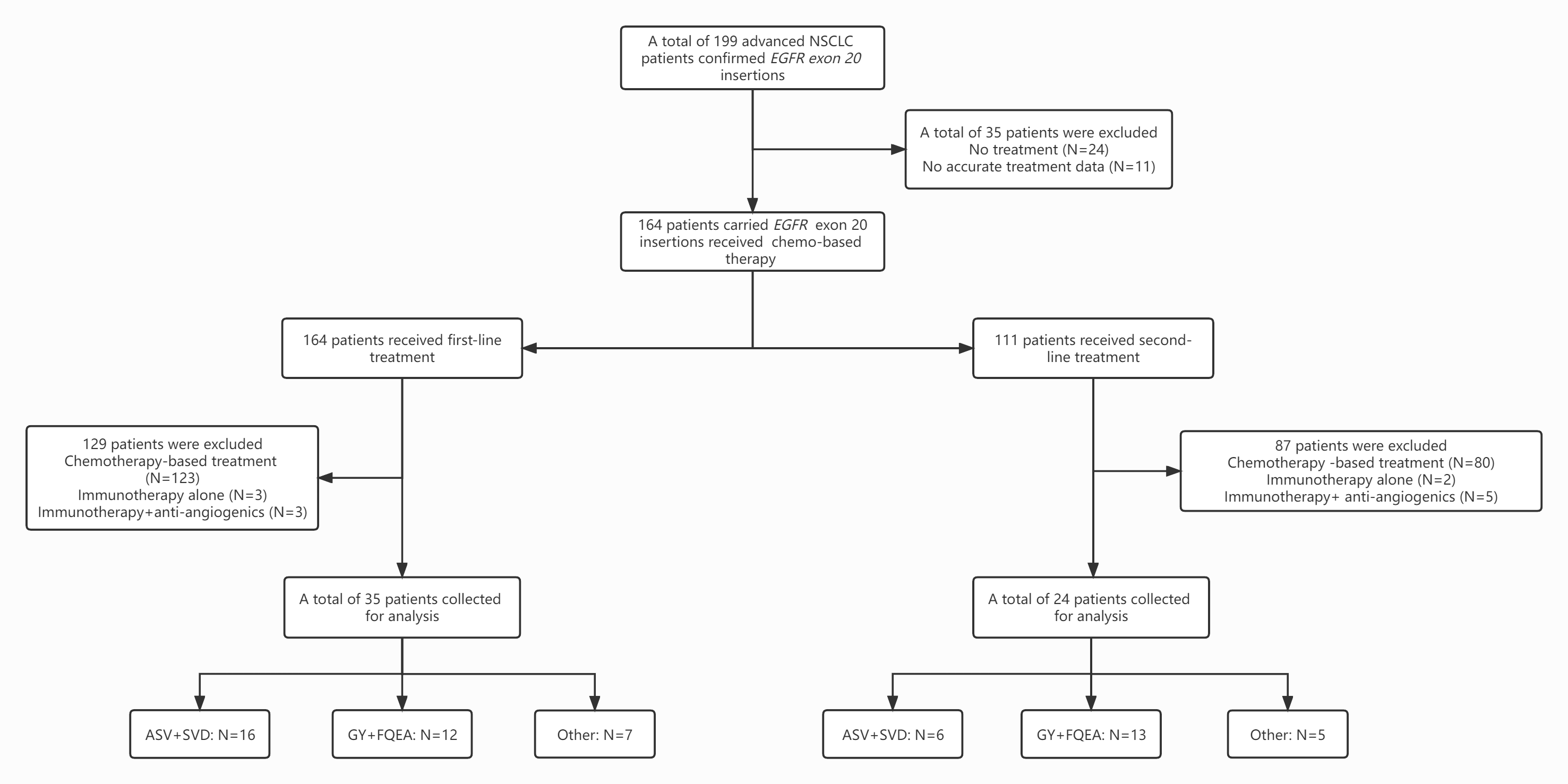

Supplement: Supplementary file 2 [file Image1.TIF]
